# COVID-19 Pneumonia Complicated by Seizure Due to Severe Hyponatremia

**DOI:** 10.7759/cureus.15603

**Published:** 2021-06-11

**Authors:** Mhd Baraa Habib, Mohammad Khair Hamad, Tarif Kalash, Ashraf Ahmed, Mouhand F Mohamed

**Affiliations:** 1 Internal Medicine, Hamad Medical Corporation, Doha, QAT; 2 Endocrinology and Diabetes, Hamad Medical Corporation, Doha, QAT; 3 Family Medicine, Hamad Medical Corporation, Doha, QAT; 4 Internal Medicine, Hamad General Hospital, Hamad Medical Corporation, Doha, QAT

**Keywords:** covid-19, seizure, post-vaccination, severe hyponatremia, siadh

## Abstract

Coronavirus disease 2019 (COVID-19) is a widespread disease. Hyponatremia in the setting of the syndrome of inappropriate antidiuretic hormone secretion (SIADH) was described with severe acute respiratory syndrome coronavirus 2 (SARS-CoV-2) infection. Nonetheless, seizure as a prominent manifestation of hyponatremia associated with COVID-19 is rare. We present a case of a middle-aged man with mild COVID-19 pneumonia, who developed a seizure due to SIADH-related severe hyponatremia.

## Introduction

Coronavirus disease 2019 (COVID-19) has spread worldwide, causing significant morbidity and mortality. The infection usually manifests in fever, cough, fatigue, nausea, diarrhea. However, a wide array of symptoms has been described [1]. Neurological manifestations were frequently reported in the context of severe acute respiratory syndrome coronavirus 2 (SARS-CoV-2) infection. These include headache, dizziness, confusion, and rarely, seizures [2]. Vaccines have been widely adopted as a strategy to prevent SARS-CoV-2 severe infection. BNT162b2 messenger ribonucleic acid (mRNA) COVID-19 vaccine is becoming widely available with an efficacy among adults of 52% and 94.6% after receiving one and two doses, respectively [3].

Hyponatremia accompanying SARS-CoV-2 infection is usually mild to moderate. It can result from low oral intake of fluids, diarrhea, diuretics, and in many instances, it has been attributed to the syndrome of inappropriate antidiuretic hormone secretion (SIADH). Symptoms of hyponatremia such as nausea, dizziness, and headache have been reported in COVID-19 [4]. However, seizure in the context of COVID-19-associated hyponatremia, to the best of our knowledge, was not previously reported.

## Case presentation

We present the case of a 53-year-old male patient, known to have hypertension, receiving perindopril and indapamide. The patient has no history of seizure disease He received the first dose of the BNT162b2 mRNA COVID-19 vaccine. Five days later, he developed a mild dry cough. COVID-19 was suspected and confirmed with polymerase chain reaction (PCR) from a nasopharyngeal swab. The physical examination was unremarkable at the time, and the chest X-ray was normal. He was admitted for fourteen days in a quarantine facility following our local protocol and was discharged on day fourteen in good condition. He was there on the usual amount of oral fluid with no significant change in his diet. On the same day of discharge, he developed a tonic-clonic seizure. As described by the bystanders, the seizure lasted for one to two minutes, and it was associated with loss of consciousness. Subsequently, he was brought to our hospital. Upon presentation, he was initially confused and drowsy but gradually regained full consciousness over the next 10-15 minutes (this was suggestive of a postictal state). Upon questioning him, he complained of mild headache, nausea, and dizziness but denied any other symptoms preceding the seizure. His blood pressure was 150/87 mmHg, and his heart rate was 91 beats per minute. He was assessed to be euvolemic by the admitting team. Laboratory tests revealed severe hyponatremia (Na = 102 mmol/L), significantly lower than his sodium level two weeks before (132 mmol/L) (Table 1).

**Table 1:** Laboratory tests upon hospital admission. NT pro-BNP: N-terminal pro-brain natriuretic peptide

Detail	Value w/Units	Normal Range
Serum Sodium	102 mmol/L	135-145 mmol/L
Random Glucose	6.7 mmol/L	3.3-5.5 mmol/L
Serum Osmolality	229 mmol/kg	275-295 mmol/kg
Urine Osmolality	293 mmol/kg	150-1,150 mmol/L
Spot Urine Sodium	53 mmol/L	Normal <20 mmol/L
Serum Cortisol	412.0 nmol/L	138-635 nmol/L
Thyroid Stimulating Hormone	0.99 mIU/L	0.30-4.20 mIU/L
Serum triglyceride	0.9 mmol/L	Normal <1.7 mmol/L
Hemoglobin	14.3 gm/dL	13.0-17.0 gm/dL
Urea	2.0 mmol/L	2.5-7.8 mmol/L
Creatinine	68 umol/L	62-106 umol/L
Potassium	3.3 mmol/L	3.5-5.3 mmol/L
Bicarbonate	25 mmol/L	22-29 mmol/L
NT pro-BNP	52.9 pg/mL	0.0-210.0 pg/mL

**Figure 1:**
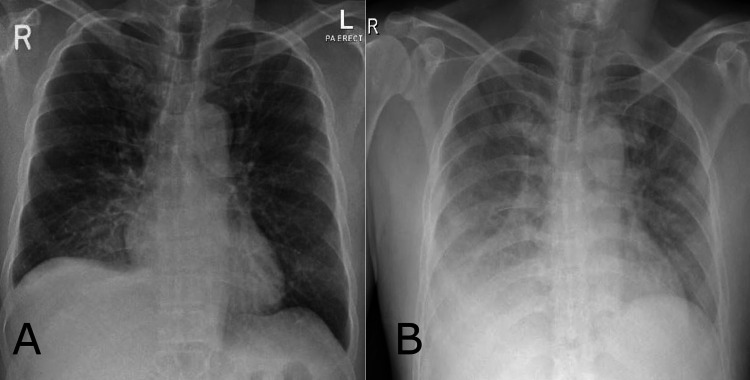
Chest X-ray: (A) prominent bronchovascular markings with ground glass opacities in the lower zone of both lungs; (B) repeated chest X-ray showing worsening reticular alveolar infiltrates involving both lungs, predominately at bases and middle quadrants.

A new chest X-ray was suggestive of bilateral pneumonia (Figure 1A). Indapamide was held, and the patient was started initially on boluses of hypertonic saline 3%. Hyponatremia work-up revealed low serum osmolality, relatively high urine osmolality, high urine sodium with normal renal function, cortisol, and thyroid function (Table 1). This made COVID-19-associated SIADH the most likely diagnosis, especially since the patient was initially euvolemic and had previously normal sodium readings while taking his chronic medications (perindopril and indapamide). Based on this, free water restriction was advised to a maximum of 800-1000 ml/day. Magnetic resonance imaging (MRI) of the head was done on day three post-admission and was unremarkable. We postulated that the unintended delay in obtaining MRI led to the resolution of expected brain edema associated with severe hyponatremia. During hospitalization, his symptoms of nausea and dizziness improved, but he had a mild, increasing dry cough with a repeated chest X-ray showing the progression of bilateral lung infiltrates (Figure 1B).

**Figure 2:**
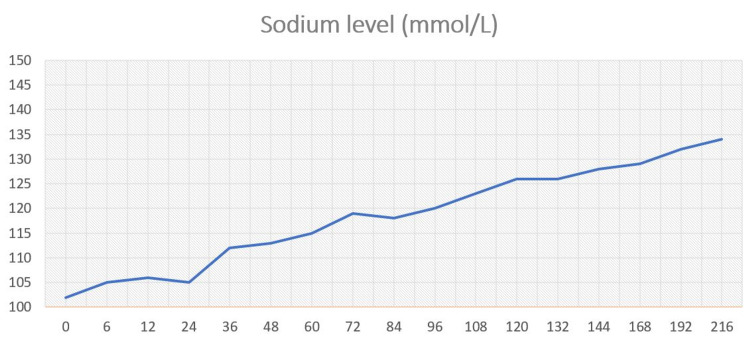
Sodium level (mmol/L) during hospitalization (in hours).

Local COVID-19 pneumonia treatment protocol was administered (favipiravir, amoxicillin, and azithromycin). The patient remained clinically stable and did not need oxygen support. Over the next few days, there was a gradual improvement in cough and serum sodium (Figure 2).

The calculated urine output was around nine liters over seven days during the recovery phase (urine was not collected properly during the first three days). Electroencephalogram (EEG) to rule out the possible COVID-19 encephalopathy was deemed unnecessary due to the rapid improvement of his symptoms, and the absence of seizure recurrence. Follow-up two weeks after discharge revealed the absence of new symptoms or symptoms recurrence, and a normal sodium level of 140 mmol/L.

## Discussion

Severe hyponatremia-induced seizure is a medical emergency associated with morbidity and mortality necessitating prompt recognition and treatment [5]. SIADH, one of the causes of hyponatremia, can be a complication of many diseases such as malignancy, pulmonary or central nervous system disorders, and can be induced by certain medications. SIADH is diagnosed by a combination of supportive laboratory tests (including serum and urine sodium level, serum and urine osmolality) and the exclusion of other causes of hyponatremia [6]. COVID-19 related hyponatremia was reported frequently in COVID-19 patients, and it could be the only presenting manifestation [4,7]. The mechanism of COVID-19-induced SIADH is not well elucidated, but it was postulated that inflammatory cytokine release [4], stress, intravascular volume depletion, lower osmolarity, and ventilation perfusion-mismatch play a role in dysregulating the antidiuretic hormone (ADH) release [8]. Many patients respond to fluids restriction and treatment of the underlying precipitant [4].

In our case, a hyponatremia-induced seizure was the most likely diagnosis given the severe hyponatremia, absence of seizure recurrence after sodium correction, and the normal brain MRI. EEG and lumbar puncture were initially planned if there had been no improvement after treating hyponatremia. The cause of hyponatremia was deemed to be SIADH associated with COVID-19 supported by laboratory tests and normal adrenal and thyroid functions (Table 1) [6]. Although limited oral intake and diuretic may in part explain hyponatremia in our case, the fact that he was on regular indapamide for two years with stable sodium levels; additionally, the euvolemic status and the simultaneous drop in sodium level with worsening SARS-CoV-2 infection supported the diagnosis of COVID-19-associated SIADH.

## Conclusions

Amidst the current pandemic, COVID-19 should be part of the differential diagnosis of hyponatremia of unclear etiology. Vice versa, COVID-19 patients should be screened for hyponatremia. While COVID-19 is linked to SIADH and hyponatremia, our case suggests that COVID-19-associated SIADH can precipitate severe symptomatic hyponatremia. Early diagnosis and treatment of hyponatremia and COVID-19 with sodium monitoring can lead to better outcomes.
